# A Bibliometric Analysis of Performance Measurement and Management in Child and Adolescent Healthcare Services

**DOI:** 10.3390/healthcare14162598

**Published:** 2026-08-18

**Authors:** Ioannis Ch. Lampropoulos, Maria Kalogera

**Affiliations:** Department of Business Administration, University of Patras, University Campus, 26504 Patras, Greece; kalogera@upatras.gr

**Keywords:** performance measurement, performance management, quality of care, quality indicators, benchmarking, children’s health services, bibliometric analysis, VOSviewer

## Abstract

**Highlights:**

**What are the main findings?**

- Keyword co-occurrence analysis of 643 Scopus documents (1990–2026) identified four thematic clusters in pediatric/adolescent healthcare performance research, with “quality of health care” emerging as the central bridging concept, while administrative/institutional terms were consistently older than clinical and operational terms.
- The retrieved literature was strongly concentrated in clinical and health-related subject areas, while explicitly administrative and managerial perspectives were comparatively underrepresented; the exact term “performance measurement” did not meet the predefined keyword inclusion threshold.

**What are the implications of the main findings?**

- Future research should examine the development and empirical evaluation of administrative and strategic performance-management approaches tailored to pediatric and adolescent healthcare, including whether frameworks such as Balanced Scorecard-style or KPI-based architectures are suitable and effective in this specific context.
- Researchers and policymakers should interpret keyword-based bibliometric patterns cautiously, as the terminology represented in a thresholded network may not fully capture the conceptual content of the field.

**Abstract:**

**Background/Objectives:** Performance measurement and management is a key administrative function; however, its application to child and adolescent healthcare services remains poorly mapped in the international literature. This study attempts a systematic bibliometric mapping of the field. To the best of our knowledge, previous bibliometric studies in pediatric and related healthcare fields have primarily focused on specific clinical or service domains, whereas the intersection of performance measurement and management with child and adolescent healthcare services has not been specifically mapped. The novelty of the present study lies in addressing this intersection through an integrated bibliometric assessment of its thematic, temporal, and geographical structure. **Methods:** A bibliometric analysis was performed on the Scopus database, with a query that combined a proximity operator (W/10) and Boolean logic, yielding 643 documents (1990–2026). After metadata cleaning and application of an occurrence threshold (≥15), 111 keywords were analyzed with VOSviewer (network, overlay, density, targeted analysis, bibliographic coupling of countries). **Results:** Research production increased strongly since 2010, peaking in 2025. Four thematic clusters emerged—quality of care, clinical outcomes, administrative framework, emergency/operational care—with the term “quality of health care” as the central hub-bridge. The administrative cluster was linked to older publications relative to the clinical/operational clusters. Country-level bibliographic coupling identified distinct geographical patterns in cited-reference similarity, including a predominantly European cluster, a transcontinental cluster dominated by the United States and Canada, and a separate Australian cluster. The term “performance measurement” itself did not meet the inclusion threshold, reflecting the methodological effect of the selected occurrence threshold rather than the absence of the concept from the literature. **Conclusions:** The retrieved literature was strongly concentrated in clinical and health-related subject areas, while explicitly administrative and managerial perspectives appeared comparatively limited. This pattern indicates a potential research gap that warrants further investigation rather than confirming the absence of managerial performance frameworks in the broader field.

## 1. Introduction

Performance measurement and management are fundamental functions of any organization that seeks to systematically monitor, evaluate and improve its operation. The roots of the field can be traced back to the early 20th century, while its conceptual maturation took place in the 1990s, when individual scientific streams—mainly operations, strategy and accounting—converged on a common understanding of the subject (Pavez et al. [1]) At the same time, since the late 1970s, dissatisfaction with traditional, purely financial and past-result-oriented measurement systems led to what has been called the “performance measurement revolution,” with the adoption of non-financial indicators—such as quality, customer satisfaction, cycle time and innovation—which functioned as leading indicators of financial performance (Nudurupati et al. [2]).

The most influential development of this period was the development of the Balanced Scorecard by Kaplan and Norton [3], which introduced a performance measurement system that combined financial indicators with three additional functional dimensions—customer satisfaction, internal processes, and organizational learning/improvement—translating the organization’s strategy and mission into specific, measurable goals. Its subsequent application to healthcare was examined by Zelman et al. [4], who reviewed the use of the Balanced Scorecard in healthcare organizations. This approach, originally designed for the private sector, was gradually adopted by health care organizations. More recent evidence indicates that integrated performance management and measurement systems continue to build on this approach by aligning organizational performance objectives and indicators with strategy and by structuring the selection and integration of performance indicators across hierarchical levels by Fradj et al. [5]. As Zelman et al. [4] point out, the Balanced Scorecard has proven relevant to the health care sector, provided it is modified to reflect the specificities of the sector by incorporating additional dimensions such as quality of care, outcomes, and access to services. A systematic review by Amer et al. [6] identified 36 applications of the Scorecard in health organizations, which resulted in 13 main performance dimensions (financial, efficiency, safety, time, patient and health staff dimensions, among others), highlighting the breadth and adaptability of the framework. At the national health system level, the implementation of a Balanced Scorecard in Afghanistan, over a five-year period (2004–2008), documented statistically significant improvements in all performance areas monitored, demonstrating the tool’s potential to function as a mechanism for systematic improvement at the health system level (Edward et al. [7]).

In this context, quality indicators are the key tools for translating performance into quantitatively measurable quantities. As reported by Ou et al. [8], quality indicators are defined as quantitative measures used to evaluate the governance, administration and delivery of health care, while a subset of them, quality metrics, function as reference points or benchmarks. More broadly, performance indicators provide measures for monitoring, assessing, and managing health-system performance across dimensions such as effectiveness, equity, efficiency, and quality by Arah et al. [9]. Performance measurement involves the assessment of healthcare processes and outcomes through performance measurement systems, which also enables comparison and benchmarking between similar aspects of care at different levels of the healthcare system by Persaud and Nestman [10]. Performance management extends beyond performance measurement by providing mechanisms through which feedback from performance indicators can be translated into strategies for action and quality improvement [10]. Importantly, the practical use of performance indicators for decision-making depends on their actionability, including their alignment with the intended purpose of use and relevant methodological, contextual, and managerial considerations by Barbazza et al. [11]. Thus, these concepts can be understood as an interconnected process: performance indicators provide the basis for measurement; benchmarking enables comparison; performance management uses the resulting information to inform action; and quality improvement uses this feedback to modify healthcare activities and assess their outcomes [9,10,11].

A related contemporary development is value-based healthcare, which emphasizes the relationship between patient outcomes and the costs required to achieve those outcomes. Recent evidence highlights leadership, integrated care organizations, the identification and standardization of outcome measures, and incorporation of the patient perspective as important elements of value-based healthcare implementation (Fernández-Salido et al. [12]).

The importance of systematic performance measurement takes on particular weight when examined in the context of child and adolescent health services. The medical and psychosocial needs of children and adolescents differ substantially from those of adults, which requires differentiated standards of care, specialized equipment, appropriately designed facilities and specially trained personnel (Hill et al. [13]). In a similar vein, Baltag and Sawyer [14] point out that health services must abandon treating adolescents as simply “older children” or “younger adults”, adopting approaches based on standards and indicators that respond to their real developmental needs. This position is also reinforced by the World Health Organization’s Global Quality Standards for Adolescent Health Services, which explicitly set an evaluation framework around dimensions such as health literacy, the appropriateness of the services provided, the capabilities of providers, and the participation of adolescents themselves in shaping care (Quinlan-Davidson et al. [15]).

At the same time, performance measurement in health services is not just a technical issue, but is directly linked to accountability, systems improvement and policy design. As Baars et al. [16] note in the context of mental health, performance measurement simultaneously serves the objectives of accountability, quality improvement and performance management, while requiring the systematic recording of structural, process and outcome indicators. At the organizational level, the role of management emerges as crucial for achieving quality care in pediatric/adolescent care. In particular, Alessandrini and Knapp [17] emphasize the need to appoint quality leaders specifically for pediatric emergency care, with responsibility for the selection of indicators, monitoring performance and implementing improvement cycles. In a related context, the qualitative study by Goff et al. [18] in high-performing pediatric primary care structures highlighted the organizational culture of quality improvement, teamwork, and the integration of the family as an improvement partner as determining organizational factors that differentiate the most efficient care structures from the rest.

Despite the theoretical and practical maturity that the field of performance management has developed in the broader health field, the literature shows a significant asymmetry regarding its application in the context of pediatric and adolescent care. Sawyer et al. [19], in one of the few attempts to develop a conceptual framework for measuring quality specifically for adolescents in a hospital setting, characteristically report that they were unable to identify any pre-existing conceptual framework or set of health care quality indicators specifically designed for adolescents in a hospital setting, highlighting the risk of applying tools developed for adults without taking into account developmental differences. A similar observation is made by Bailey et al. [20], who note that quality measurement in pediatrics “has evolved at a slower pace” compared to adult care, partly due to the limited transferability of concepts and indicators (such as mortality or readmissions) from the adult to the pediatric context, as well as due to substantial differences in quality objectives between the two populations.

This observation becomes particularly important when combined with the fact that performance measurement and management, as a distinct scientific field, has its roots in management science and accounting—and not in clinical sciences. Recent research also illustrates the continuing development of healthcare-specific KPI systems. For example, a 2024 systematic review by Magedanz et al. [21] of KPIs for clinical pharmacy services identified 225 indicators across 13 studies, predominantly process indicators, but reported limited methodological quality, inadequate evidence support, lack of piloting or validation, and the absence of benchmarking reference values. However, as highlighted by the literature review so far, the application of performance management tools (such as the Balanced Scorecard or Key Performance Indicators systems) in the context of pediatric/adolescent care remains fragmented, while the majority of available quality assessment frameworks in this area remain strictly clinically oriented, without substantial integration of administrative/managerial performance dimensions (strategy, cost, resource efficiency, organizational capacity). This finding may reflect a potential research gap, which the present study attempts to systematically investigate. To map this field, this study adopts the method of bibliometric analysis. The bibliometric approach is an appropriate methodological choice for the study of broad, interdisciplinary and rapidly developing research fields, as it allows for the objective, quantitative organization of a large volume of literature, the identification of thematic clusters and research trends, as well as the mapping of collaboration networks between countries and research groups. Tools such as the VOSviewer software (version 1.6.20) have been established in the international literature as being particularly effective for the construction of readable visual maps of keywords, co-authorship and bibliographic linkage, facilitating both researchers and policymakers in understanding the evolution and structure of a scientific field.

Bibliometric approaches have previously been applied to specific pediatric and related healthcare domains. For example, Zhang and Li [22] used bibliometric methods to examine research focuses and trends in pediatric palliative care, while Wang et al. [23] mapped the scientific output and global research trends in perinatal palliative care using bibliographic coupling, co-authorship, and keyword co-occurrence analyses. These studies demonstrate the application of bibliometric methods to specific pediatric and perinatal clinical fields; however, to the best of our knowledge, no previous bibliometric study has specifically mapped the intersection of performance measurement and management with child and adolescent healthcare services. The present study addresses this gap by examining the thematic, temporal, and geographical structure of this literature.

Based on the above, this study poses the following research question: How has the international literature on performance measurement and management in child and adolescent health services evolved, and what are the dominant thematic, temporal and geographical patterns that characterize it? The study is exploratory and descriptive in nature and does not test a predefined hypothesis or causal scientific claim; rather, it aims to map and characterize the structure and development of the retrieved literature through bibliometric analysis. To answer this question, a systematic bibliometric analysis of data from the Scopus database was carried out, using keyword co-occurrence, temporal overlay, density and country bibliographic coupling techniques, as presented in detail in the following sections.

## 2. Materials and Methods

This study applies bibliometric analysis to systematically map the international literature on performance measurement and management in the context of child and adolescent health services. The bibliographic data were drawn from the Scopus database, which is widely recognized for its broad interdisciplinary coverage and full compatibility with bibliometric analysis tools, making it particularly suitable for mapping research trends in health issues.

The search was performed in the TITLE-ABS-KEY fields (title, abstract, keywords) with the following query, which was designed to capture the intersection of three conceptual dimensions: (a) performance measurement and management terminology, (b) the pediatric/adolescent population, and (c) the health services context:

TITLE-ABS-KEY ((“performance measurement” OR “performance management” OR “performance indicator*” OR “performance evaluation” OR “performance assessment” OR “key performance indicator*” OR “quality indicator*” OR benchmarking) W/10 (pediatric* OR paediatric* OR child* OR adolescent*)) AND TITLE-ABS-KEY (“health service*” OR healthcare OR “health care” OR “health system*” OR hospital* OR “clinical service*”).

In this search, a proximity operator (W/10) was applied between the terms related to performance and the terms related to the target population, instead of a simple Boolean AND. The use of proximity operators (e.g., NEAR/n, ADJ/n, W/n) is recognized in the methodological literature as a means of improving the precision of a search, ensuring that relevant terms appear in substantial conceptual proximity and not simply anywhere in the same text [24]. A similar practice has been applied in other systematic reviews, where the use of proximity operators (e.g., ADJ12/NEAR12) was specifically adopted to reduce false positives (Camp et al. [25]). At the same time, the relevant PRESS methodological guideline [26] points out that choosing a proximity operator over AND can improve accuracy, while recommending careful weighting of the width of the proximity window (e.g., W/n), as excessively narrow windows may limit the sensitivity (recall) of the search, excluding possible relevant wording variations. Preliminary query testing indicated that the use of the W/10 proximity operator substantially reduced the volume of retrieved literature compared with broader Boolean formulations and was therefore selected as a pragmatic means of increasing search specificity while preserving conceptual proximity between performance-related and pediatric/adolescent terms. The preliminary comparison was based on retrieval volume and inspection of search results rather than on a formal precision–recall assessment or title-and-abstract eligibility screening. Accordingly, the choice of W/10 should be understood as a search-design decision intended to improve specificity, rather than as an empirically validated optimal threshold for precision and sensitivity.

The search was conducted on 23 June 2026 and yielded 643 records, which were exported directly from Scopus in CSV format with all available metadata fields selected. No additional filters (document type, language, subject area) were applied at this stage, in accordance with standard practice in bibliometric analyses, which favors comprehensive data retrieval in order to minimize the risk of omitting relevant studies—a similar approach (absence of additional exclusion filters) was also followed by Lampropoulos et al. [27].

Of the 643 documents retrieved, the vast majority corresponded to research articles (n = 545, 84.8%) and review articles (n = 56, 8.7%), while other types of publications (conference papers, book chapters, notes, editorials, etc.) represented a total of only 6.5% of the total. Regarding language, 607 records were published in English, 13 in Spanish, 10 in German, 3 in Chinese, 3 in Russian, and one record each in Czech, Dutch, French, Norwegian, Persian, Portuguese, and Serbian. As the dataset originated from a single Scopus search, no document-level deduplication procedure was applied prior to the bibliometric analysis. No documents were subsequently excluded on the basis of document type, language, or subject area; therefore, the full set of 643 retrieved records was retained for the bibliometric analysis. The extracted metadata was subjected to a review and cleaning process, which included the identification and correction of different spellings of identical terms and keywords, in order to improve the accuracy of bibliometric mappings. It is noted that the cleaning process exclusively concerned harmonization of lexical variants and conceptually related terms and the removal of general or thematically uninformative keywords via a thesaurus file in VOSviewer. The thesaurus was jointly developed and reviewed by both authors, and all term-merging and exclusion decisions were made by consensus; no disagreements requiring further resolution occurred. Term harmonization was primarily based on lexical variants, singular/plural forms, and terms considered conceptually equivalent for the purposes of the present bibliometric mapping. In a limited number of cases, closely related indicator terminology was consolidated under a broader performance-related label; for example, “performance indicators”, “key performance indicator”, and “clinical indicator” were harmonized under “performance indicator”. Such harmonization decisions were intended to reduce terminological fragmentation within the network and should not be interpreted as implying that these terms are conceptually identical in all contexts. No exclusion of documents was performed from the original dataset of 643 publications, which was kept intact throughout the analysis. The data were then analyzed using VOSviewer version 1.6.20 (Centre for Science and Technology Studies, Leiden University, Leiden, The Netherlands), through keyword co-occurrence techniques, as well as overlay visualization and density visualization, according to established methodological practices in the field (Van Eck & Waltman [28,29]). For the keyword co-occurrence analysis, a map based on bibliographic data was created from the Scopus CSV export, using all keywords (i.e., both author keywords and indexed keywords) as the unit of analysis and the full counting method. The thesaurus file described above was applied during map construction. No manual modifications were made to the VOSviewer normalization or clustering parameters; the software’s default settings were retained for network normalization and cluster construction. Network, overlay, and density visualizations were subsequently generated from the resulting keyword co-occurrence network. To enhance the reproducibility of the analysis, the complete thesaurus file used for term harmonization and exclusion of thematically uninformative terms is provided as Supplementary File S1. In addition, the original VOSviewer network export containing the final keyword set used in the primary co-occurrence analysis (minimum occurrence threshold ≥ 15), together with the associated network and bibliometric attributes, is provided as Supplementary File S2. The complete VOSviewer country-level bibliographic coupling network export is additionally provided as Supplementary File S3, including the country-level network attributes and bibliographic coupling links used in the analysis. The targeted co-occurrence visualizations did not represent independently recalculated networks; rather, they were generated by selecting central terms within the original co-occurrence network and visualizing their existing links with the other nodes in that network.

**Figure 1:**
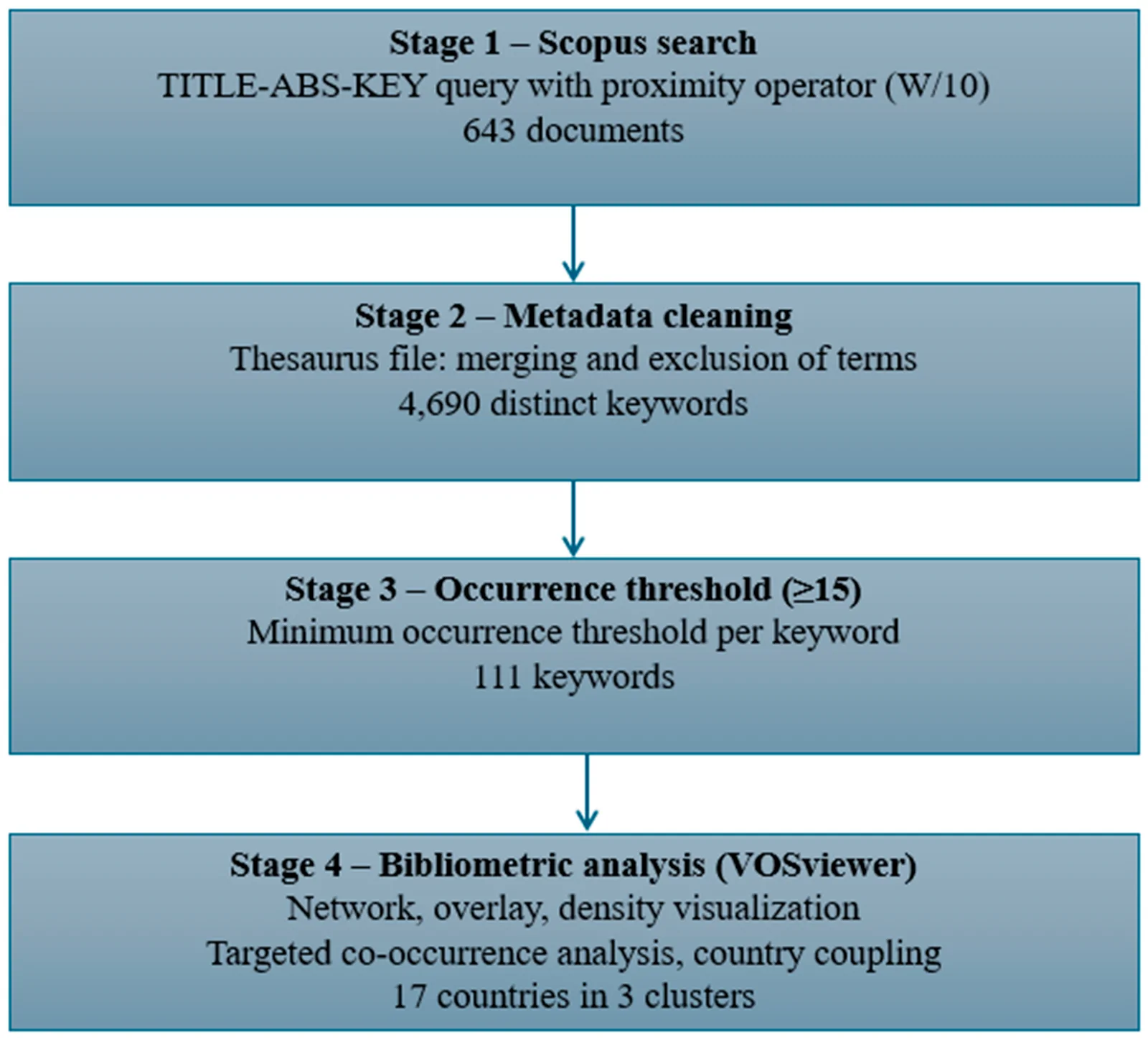
Flowchart of the methodological process.

The overall data collection and bibliometric analysis process followed in this study is schematically summarized in Figure 1.

The process included four consecutive stages. In the first stage, a search was performed in the Scopus database (TITLE-ABS-KEY fields) using a query that combined a proximity operator (W/10) and Boolean logic, yielding 643 documents. In the second stage, the extracted metadata were subjected to a check and cleaning through a thesaurus file, which merged conceptually identical terms and excluded thematically unrelated terms, resulting in a total of 4690 distinct keywords. In the third stage, a minimum occurrence threshold (≥15 per keyword) was applied, a criterion that was met by 111 keywords, which formed the basis of the co-occurrence analysis. To assess the sensitivity of the network structure to the selected keyword occurrence threshold, the co-occurrence analysis was additionally repeated using minimum occurrence thresholds of ≥10 and ≥20, while maintaining the same dataset, thesaurus file, and analytical settings. These supplementary analyses were used as sensitivity checks, while the threshold of ≥15 remained the primary analysis presented in the study. In the fourth and final stage, all bibliometric analyses were carried out through the VOSviewer software (Network, Overlay, Density visualization, targeted co-occurrence analysis of central terms, and bibliographic coupling analysis of countries), the results of which are presented in detail in Section 3.

## 3. Results

### 3.1. Descriptive Characteristics

Of the 643 documents retrieved, the vast majority corresponded to research articles (n = 545, 84.8%) and review articles (n = 56, 8.7%), while other types of publications (conference papers, book chapters, notes, editorials, etc.) represented a total of only 6.5% of the total.

**Figure 2:**
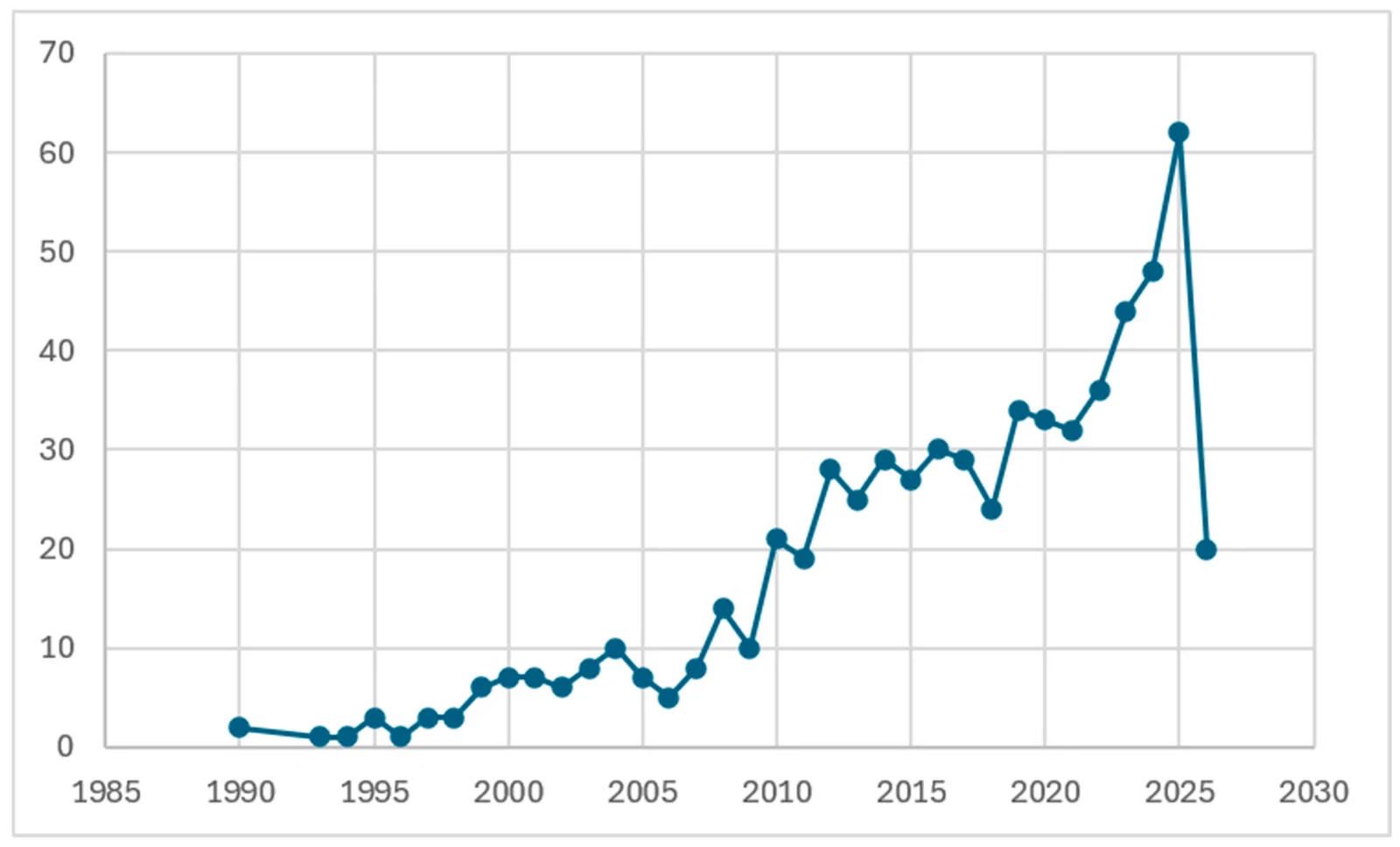
Annual scientific production of the 643 Scopus-indexed documents included in the bibliometric analysis, covering the period 1990–2026. The 2026 publication count reflects an incomplete year, as data were retrieved in June 2026.

The 643 retrieved publications span the period from 1990 to 2026, showing a clear upward trend in research production in recent years. Publishing activity remained limited until the early 2000s, gradually intensified from 2010 onwards, and peaked in 2025 (62 publications), with 2026 already showing 20 publications despite the fact that the year has not yet been completed at the time of data extraction (Figure 2). Accordingly, the 2026 publication count is reported descriptively and is not directly compared with the output of complete publication years.

This pattern suggests an ever-increasing research interest in measuring and managing performance in child and adolescent health services, particularly over the last decade.

In terms of distribution by Scopus subject area, the dataset was dominated by Medicine (551 documents) and Nursing (81), followed by Social Sciences (21), Health Professions (19), and Biochemistry, Genetics and Molecular Biology (19), whereas Business, Management and Accounting accounted for only 3 documents. These subject-area classifications provide a descriptive indication of the disciplinary distribution of the retrieved literature but should not be interpreted as a direct classification of the substantive content of individual publications, as Scopus subject categories may reflect journal-level indexing. Moreover, the limited representation of Business, Management and Accounting may partly reflect the health-focused construction of the search strategy. Accordingly, these findings indicate a possible, rather than confirmed, underrepresentation of explicitly administrative and managerial perspectives in the retrieved literature. It is noted that the sum of publications per subject area exceeds the total number of 643 documents, as in the Scopus database a document can be classified simultaneously in more than one subject area.

**Figure 3:**
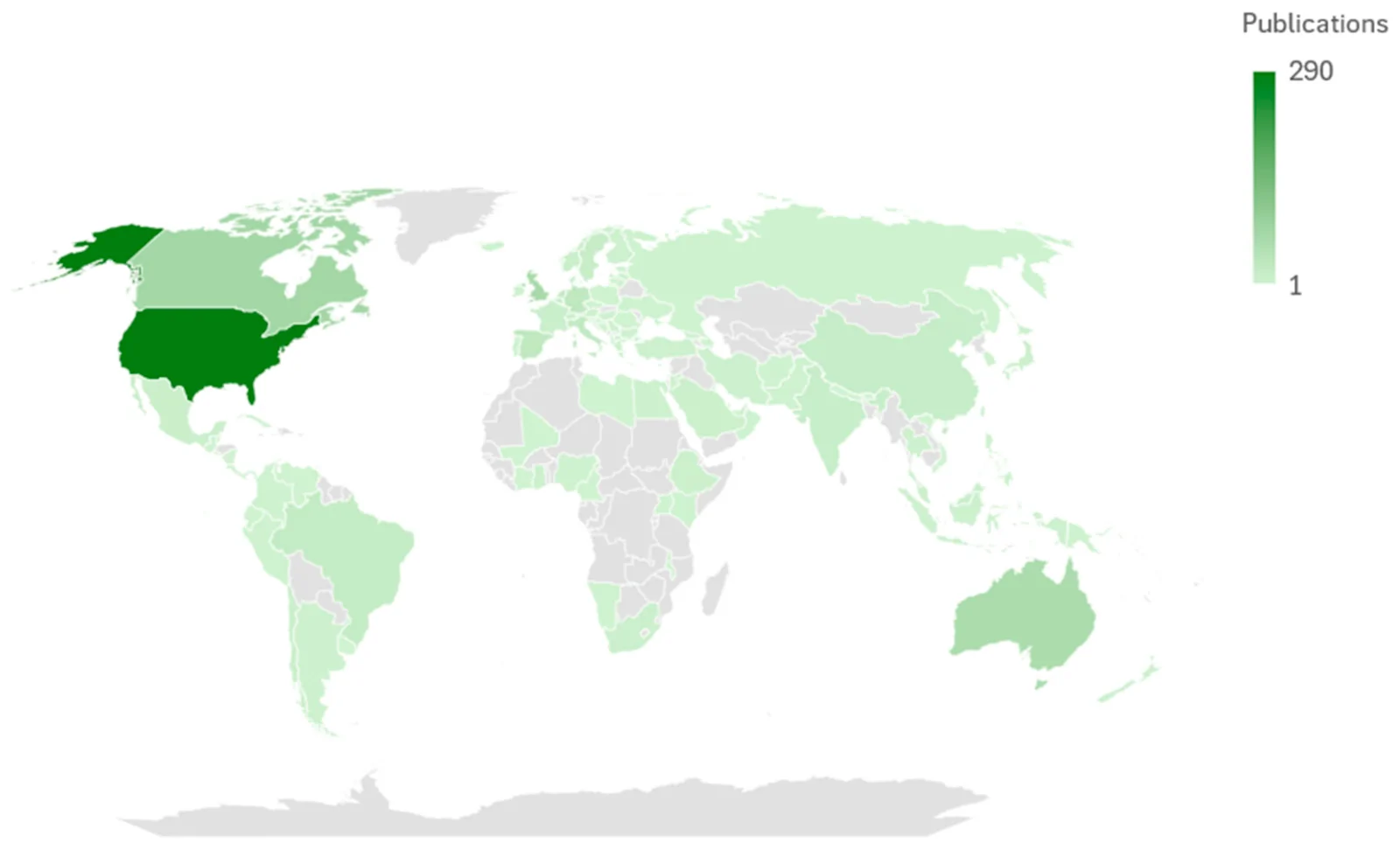
Distribution of publications by country.

In terms of geographical distribution (Figure 3), the United States holds the first place with 290 publications, followed by Canada (70), the United Kingdom (63) and Australia (57)—these countries collectively account for the largest portion of the data set. These publication counts were obtained directly from the Scopus descriptive country/territory analysis.

The European contribution is scattered across many countries (Germany, Spain, Italy, the Netherlands, Sweden, etc.), while emerging research activity is also observed in countries such as Brazil, China, India, as well as in several countries in the Middle East and Africa, which indicates a growing international engagement with the topic, although with a clear concentration in English-speaking health systems.

### 3.2. Keyword Co-Occurrence Analysis (Network Visualization)

Before constructing the keyword co-occurrence map, a thesaurus file was implemented in the VOSviewer software, which served two purposes: (a) merging conceptually identical terms that appeared in different forms of writing (e.g., singular/plural, with or without MeSH-style terms), such as “quality indicators” and “quality indicators, health care” into the single “quality indicator”, or “performance indicators” and “clinical indicator” into the single “performance indicator”, and (b) excluding terms that were deemed irrelevant to the subject of the study, as they did not represent thematic content but concerned methodological study types (e.g., “randomized controlled trial”, “cross-sectional study”), demographic characteristics (e.g., “age”, “gender”, “ethnic groups”), geographical country designations (e.g., “united states”, “germany”), and specific clinical entities/drugs outside the direct interest of the study (e.g., “amoxicillin”, “sepsis”, “pneumonia”).

After applying the thesaurus, the total number of distinct keywords was 4690 (compared to a higher initial number before mergers and exclusions). A minimum number of occurrences of 15 per keyword was then set, a criterion that was met by 111 keywords, which were included in the final keyword co-occurrence analysis.

A sensitivity analysis was additionally performed using alternative minimum occurrence thresholds of ≥10 and ≥20, while maintaining the same dataset, thesaurus file, and analytical settings. At the lower threshold of ≥10, 171 keywords were included and the network was organized into six clusters. At the higher threshold of ≥20, 68 keywords were included and the network was organized into four clusters. Compared with the primary threshold of ≥15 (111 keywords, four clusters), the higher threshold retained the same number of clusters, whereas the lower threshold produced a more granular six-cluster solution. These findings indicate that the number of identified clusters is partly sensitive to the keyword occurrence threshold, particularly when less frequently occurring terms are included.

**Figure 4:**
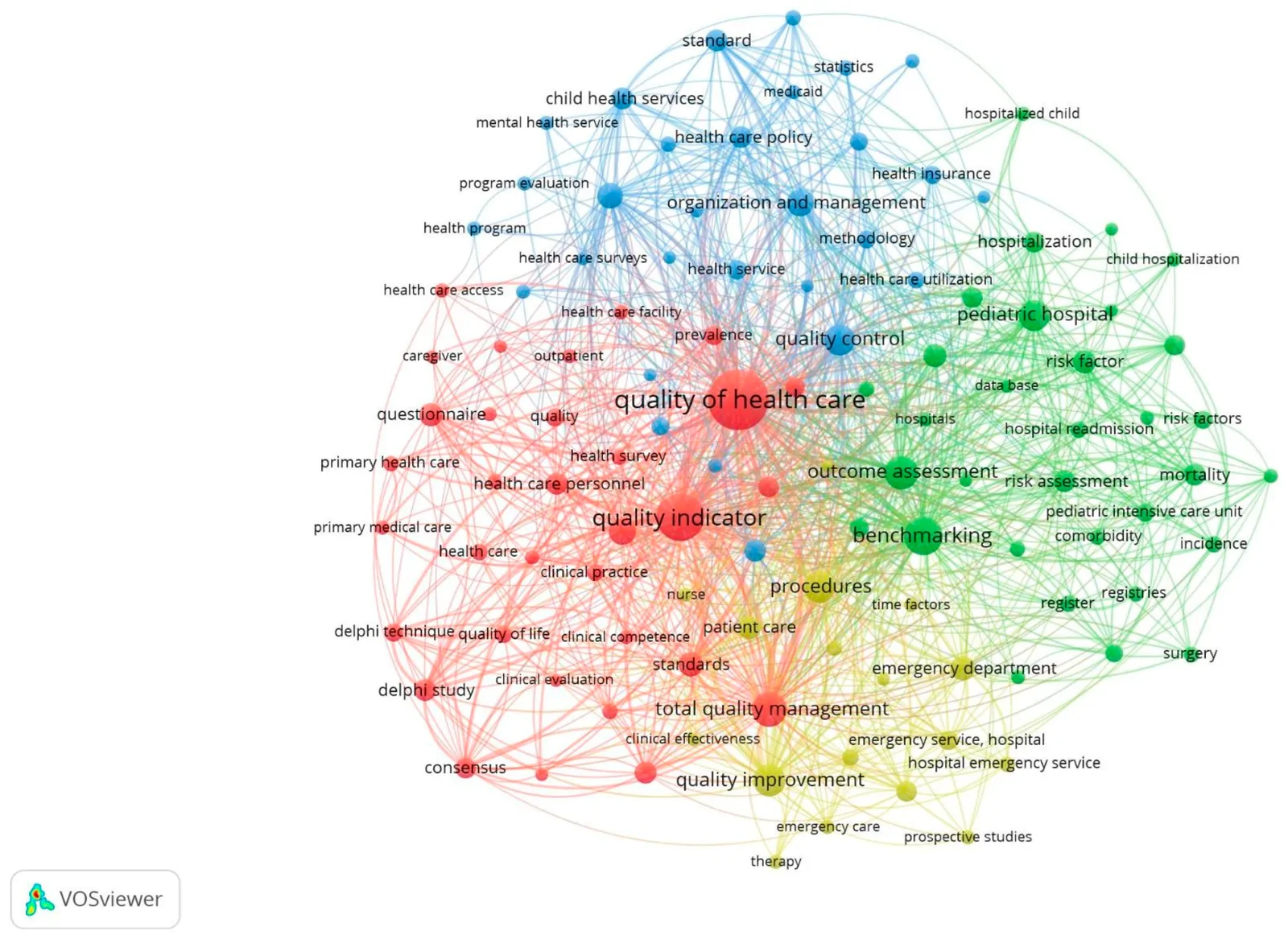
Keyword co-occurrence network (111 keywords, 4 clusters).

Keyword co-occurrence analysis, after applying the thesaurus and the minimum occurrence threshold (15), yielded a network of 111 keywords, organized into four distinct thematic clusters. This grouping reveals four clear research axes in the field of performance measurement and management in child and adolescent health services (Figure 4).

Cluster 1 (red, 33 terms)—Quality of care, standards and methodological assessment tools.

The dominant node is the term “quality of health care” (299 occurrences, total link strength 2015), followed by “quality indicator” (188, 1298) and “total quality management” (97, 777). This cluster links quality of care with methodological consensus and assessment tools, such as “practice guideline” (64), “delphi study” (39) and “delphi technique” (28), as well as with terms related to primary care (“primary health care”, “primary medical care”) and patient satisfaction/experience (“patient satisfaction”, “quality of life”, “health survey”). The average publication period of the cluster’s central terms (~2015–2017) indicates a more “mature” thematic core, consistently present in the literature.

Cluster 2 (green color, 32 terms)—Clinical outcomes, risk and hospital care.

The central term is “benchmarking” (124, 789), followed by “outcome assessment” (92, 696) and “pediatric hospital” (78, 497). This cluster gathers terms related to clinical outcomes and impacts (“treatment outcome”, “mortality”, “hospital mortality”), risk factors (“risk factor/s”, “risk assessment”), and hospital experience (“hospitalization”, “hospital readmission”, “intensive care unit”, “pediatric intensive care unit”). It is the cluster with the highest concentration of recent terms (e.g., “clinical outcome” with a mean year of 2023.3, “surgery” with 2020.5), indicating emerging interest.

Cluster 3 (blue color, 29 terms)—Administration, health policy and organizational framework.

The dominant term is “quality control” (81, 626), followed by “organization and management” (60, 442) and “health care delivery” (57, 442). This is the cluster that most directly represents the administrative/management dimension of the topic, with terms such as “health care policy” (39), “health insurance” (27), “health care planning” (20), “government” (21), “economics” (17) and “program evaluation” (19). It is also the cluster with the oldest average publication terms (e.g., “standard” 2006.6, “organization and management” 2011.8, “quality control” 2012.3), suggesting that the administrative/policy context is associated with relatively older publications compared to the other thematic clusters.

Cluster 4 (yellow color, 17 terms)—Urgent care and operational procedures.

This is the smallest in terms but thematically coherent cluster, with central terms being “procedures” (86, 596) and “quality improvement” (85, 672), linked to emergency care (“emergency department” 49, “emergency health service” 37, “hospital emergency service” 21, “emergency care” 18) and operational indicators (“scoring system”, “time factors”, “diagnosis”). It presents the most recent average publication terms across the four clusters (e.g., “hospital emergency service” 2021.5, “therapy” 2021.2, “diagnosis” 2020.8), highlighting an emerging trend of linking quality improvement to operational/emergency care.

The main characteristics of the four thematic clusters are summarized in Table 1.

### 3.3. Overlay Visualization

**Figure 5:**
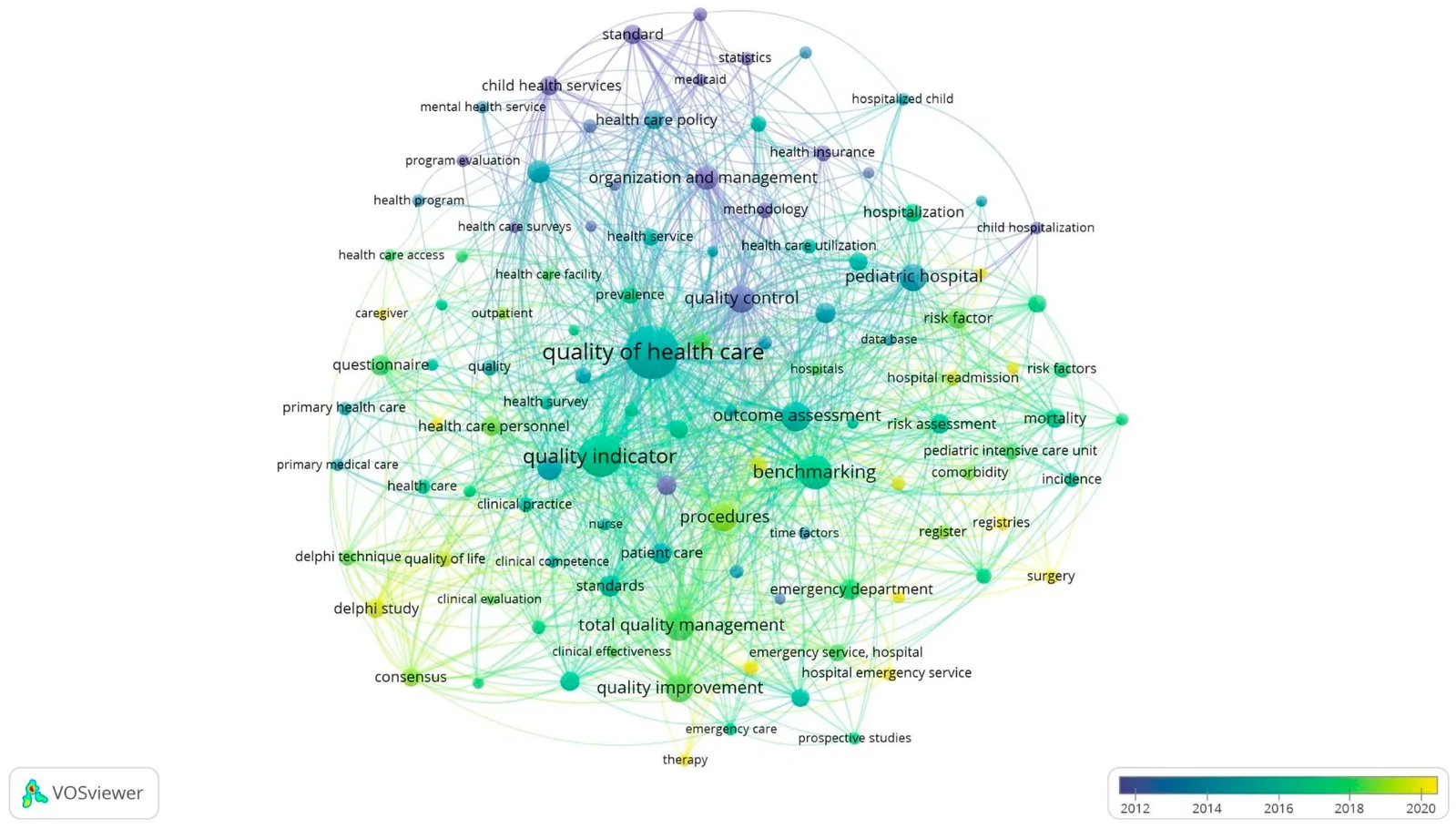
Overlay visualization of keyword co-occurrence based on average publication year.

The overlay visualization captures the temporal dimension of the co-occurrence network, assigning each keyword a color coding based on the average publication year of the relevant documents, with a scale from dark blue (older terms, ~2012) to yellow (recent terms, ~2020 and later), (Figure 5).

The analysis of the mean publication years by cluster indicates differences in the temporal distribution of keywords across the four thematic clusters. For descriptive comparison at the cluster level, the average publication year of each cluster was calculated as the unweighted arithmetic mean of the VOSviewer “Avg. pub. year” scores of all keywords assigned to that cluster. Keyword occurrence counts were not used as weights in this calculation. These cluster-level averages are therefore descriptive summaries of the temporal distribution of the keywords and are not inferential statistics. Cluster 3 (administration, health policy, organizational context) displays the oldest average publication year (2012.8), compared with the other three clusters, which range between 2017.0 and 2017.2. This temporal divergence is visually reflected in the overlay graph, where the core terms of Cluster 3 (e.g., “organization and management”, “quality control”, “health care policy”) appear in shades of blue/light blue, in contrast to the “warmer” (greenish to yellow) tones that dominate the terms of the remaining clusters.

More specifically, the 15 oldest terms in the network (average year 2006.6–2012.7) are dominated exclusively by Cluster 3 terms: “standard” (2006.6), “medicaid” (2008.6), “statistics” (2009.3), “methodology” (2010.0), “health insurance” (2010.4), “child health services” (2010.5), “quality assurance, health care” (2011.2), “organization and management” (2011.8) and “quality control” (2012.3). This finding seems to be associated with relatively older publications compared to the other thematic clusters.

In contrast, in the 15 most recent terms (average year 2019.3–2023.3) an increased presence of clinical and operational topics is observed, with a dominance of terms from Clusters 2 and 4: “clinical outcome” (2023.3—the most recent term in the network), “hospital emergency service” (2021.5), “therapy” (2021.2), “surveys and questionnaires” and “caregiver” (2020.9), “surgery” (2020.5), “registries” and “age distribution” (2020.1), “prediction” (2019.7), “tertiary care center” (2019.6), and “delphi study” (2019.4). It is also observed that terms related to emergency care (Cluster 4) and clinical outcomes/hospital experience (Cluster 2) tend to be concentrated at the recent end of the time scale, while the methodological quality core (Cluster 1) remains consistently present throughout the time span, with an average of 2017.1.

Overall, the overlay analysis indicates that terms related to administrative and institutional dimensions are associated with relatively older publications, whereas terms related to clinical outcomes and emergency care are associated with more recent publications. Indicative examples of terms with their corresponding average publication time are presented in Table 2.

### 3.4. Density Visualization

**Figure 6:**
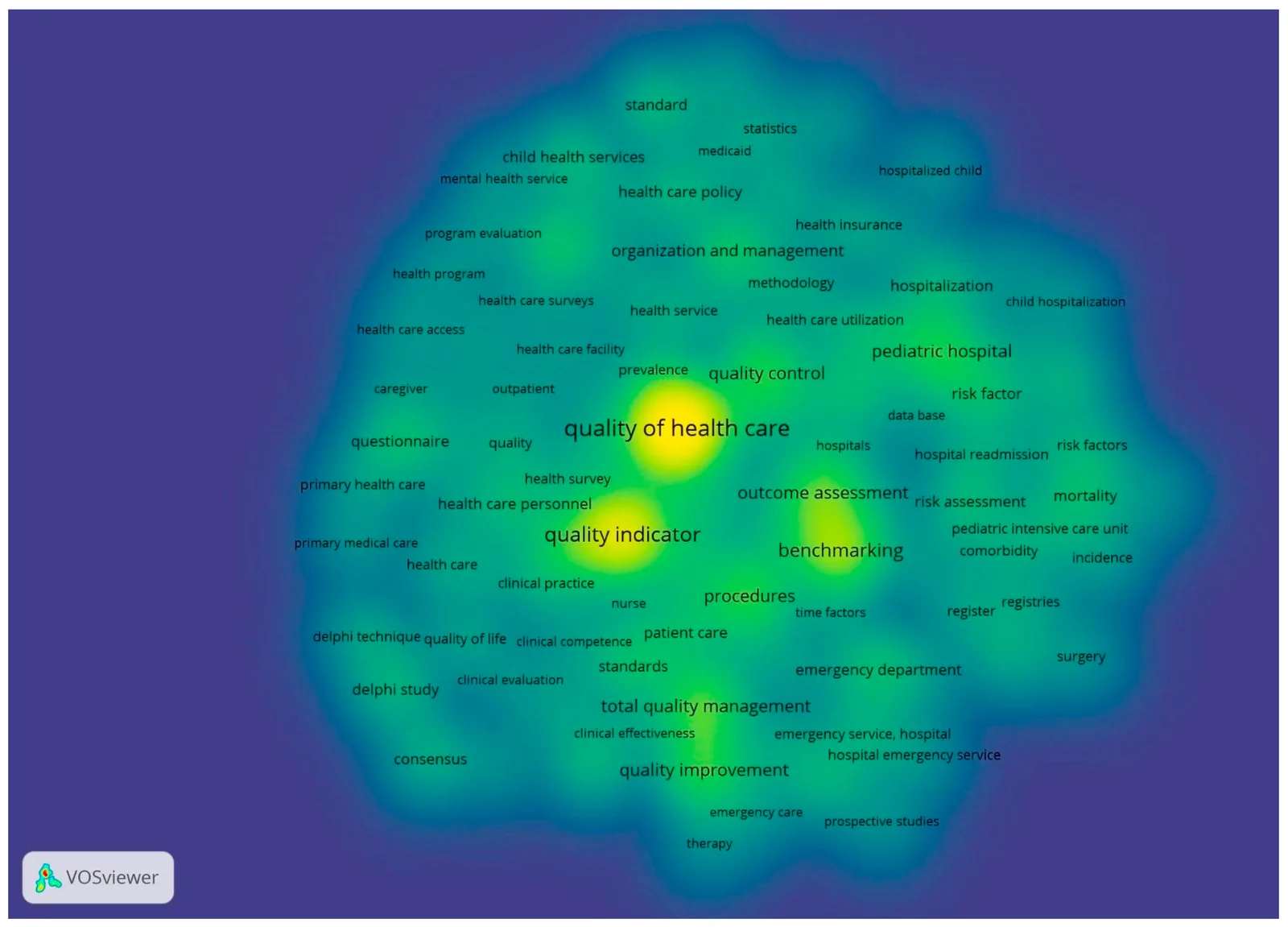
Density visualization of the keyword co-occurrence network. Higher-density areas indicate a greater concentration and weight of interconnected keywords, with the highest-density region centred on “quality of health care” and “quality indicator”.

Density visualization reveals the areas of the network with the greatest concentration and weight of terms, through a color scale from dark blue (peripheral, low density) to bright yellow (high density core), (Figure 6).

The analysis clearly confirms that the absolute thematic core of the field is concentrated around the terms “quality of health care” (299 occurrences) and “quality indicator” (188 occurrences), which form the brightest (yellow) zone of the map. This numerically confirms what is visually captured: these two terms are not simply the most frequent in the network, but function as the central conceptual “nodes-bridges” that connect all the individual thematic clusters to each other.

Around this core, a broader zone of medium-high density (green-yellow shades) develops, which includes secondary but strongly related terms such as “benchmarking” (124), “outcome assessment” (92), “organization and management” (60), “quality control” (81), “total quality management” (97), “quality improvement” (85) and “pediatric hospital” (78). This zone extends horizontally in the center of the map, indicating that quality/performance measurement functions as a unifying axis between the administrative dimension (left-top) and the clinical/operational dimension (right-bottom) of the field.

In contrast, at the periphery of the map (dark blue, low density) are terms with lower frequency of occurrence and more limited connections, such as “standard” (43), “hospitalized child” (17), “child hospitalization” (18), “registries” (21), “surgery” (20), “therapy” (17), “prospective studies” (18), and “consensus” (35). Although these terms remain thematically relevant, their peripheral position suggests either a more specialized/fragmentary role within the literature, or emerging subtopics that have not yet developed strong connections with the central core.

The Density and Network visualization comparison demonstrates that high density does not always correspond to a single thematic category (cluster), but reflects the overall connectivity of a term with the entire network. A typical example is “benchmarking” (Cluster 2) and “quality control” (Cluster 3), which, although belonging to different clusters, are both located in high density zones, functioning as nodal points of interconnection between the administrative and clinical/operational dimensions of performance in child and adolescent health services.

### 3.5. Targeted Co-Occurrence Analysis

**Figure 7:**
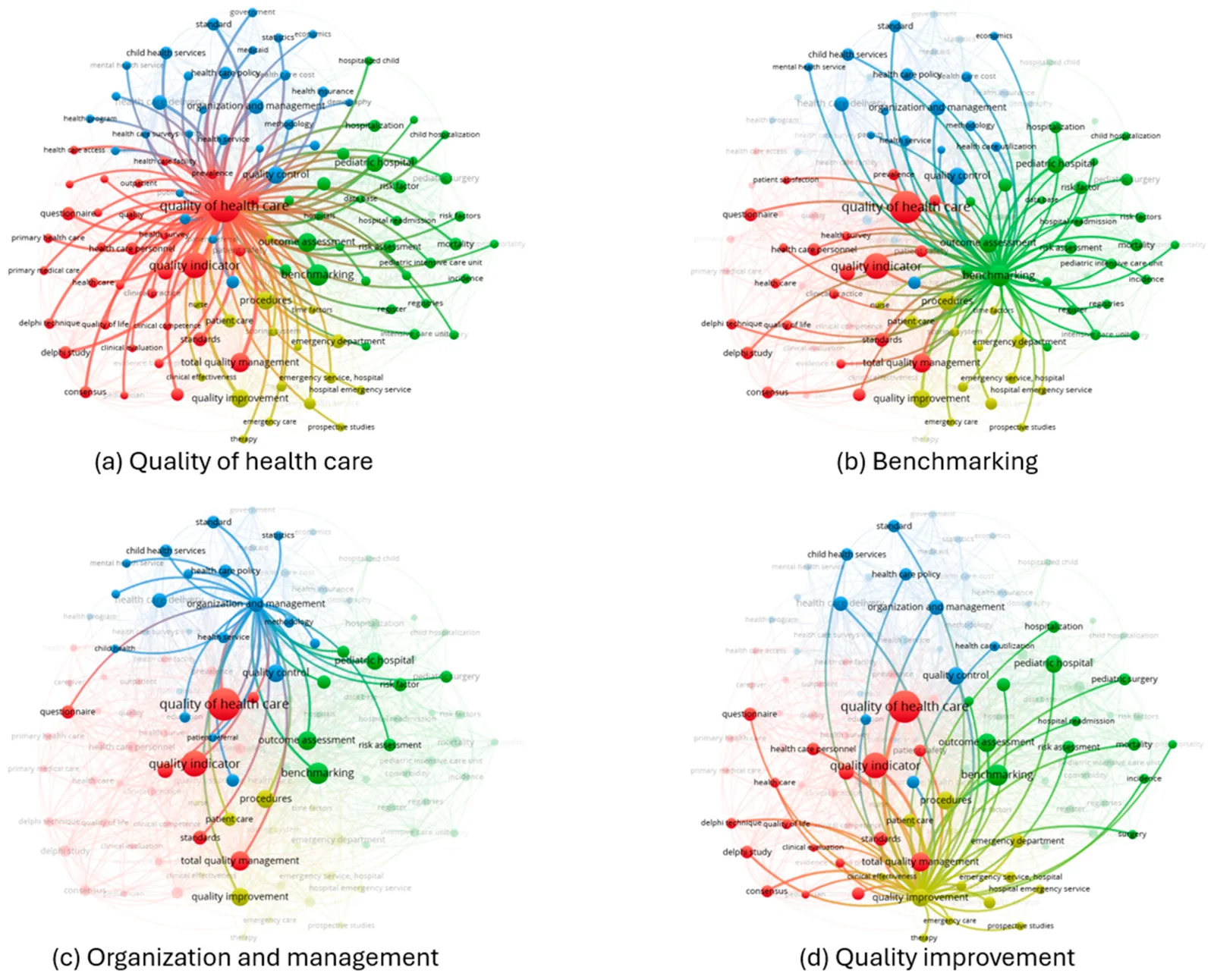
(**a**–**d**) Targeted co-occurrence maps centred on four representative keywords from the four thematic clusters.

To delve deeper into the relationships of the key conceptual nodes of the network, a targeted co-occurrence analysis was performed around four central terms, one from each thematic cluster: “quality of health care” (Cluster 1), “benchmarking” (Cluster 2), “organization and management” (Cluster 3) and “quality improvement” (Cluster 4). The selection of these terms was based on their central role within the respective cluster, as well as their thematic proximity to the key concepts of the title of this study (performance measurement and management), Figure 7.

The term “quality of health care” emerges as the most interconnected node of the entire network, functioning as a bridge between all four clusters. It is directly linked to methodological assessment tools (“delphi study”, “delphi technique”, “consensus”, “questionnaire”, “health survey”), to the administrative dimension (“organization and management”, “quality control”, “health care policy”), to the clinical/hospital dimension (“pediatric hospital”, “hospitalization”, “risk factor”, “outcome assessment”, “benchmarking”) and to operational improvement (“quality improvement”, “total quality management”, “procedures”, “patient care”). Its centrality and extensive connectivity confirm its role as a conceptual core on which all the individual dimensions of performance in child and adolescent health services converge.

The term “benchmarking” shows a strong concentration of connections mainly within its own cluster (clinical outcomes/hospital care), with terms such as “outcome assessment”, “pediatric hospital”, “risk factor/assessment”, “mortality”, “hospital readmission”, “pediatric intensive care unit”, “registries” and “incidence”. At the same time, it maintains strong connections with the central quality terms (“quality of health care”, “quality indicator”) and with the administrative/operational dimension (“organization and management”, “quality control”, “total quality management”, “quality improvement”, “procedures”). This highlights “benchmarking” as the tool that functions par excellence as a link between clinical outcome measurement and administrative/comparative performance evaluation—a concept that directly corresponds to the “performance measurement and management” of the study title.

The term “organization and management” gathers its densest connections within the administrative/institutional cluster, with terms such as “standard”, “child health services”, “health care policy”, “health care delivery”, “methodology”, “statistics”, “economics” and “medicaid”. At the same time, it extends strong connections to the clinical dimension (“pediatric hospital”, “risk factor”, “quality control”) and to the core of quality (“quality of health care”, “quality indicator”, “outcome assessment”, “benchmarking”), as well as to operational improvement (“total quality management”, “quality improvement”, “procedures”, “patient care”). Its position as a hub-bridge between the administrative/institutional framework and the other dimensions of the field confirms the role of administration as an organizational axis on which performance measurement in child and adolescent health services is inscribed.

The term “quality improvement” is closely linked to terms of implementation and operational improvement (“total quality management”, “standards”, “clinical effectiveness”, “consensus”, “delphi study”, “procedures”, “patient care”), as well as to emergency care (“emergency department”, “emergency service, hospital”, “hospital emergency service”, “emergency care”, “prospective studies”, “therapy”, “surgery”). At the same time, it maintains direct connections with the quality core (“quality of health care”, “quality indicator”) and with the administrative/clinical dimension (“organization and management”, “quality control”, “benchmarking”, “outcome assessment”, “pediatric hospital”, “hospitalization”). This connection reveals “quality improvement” as the term that most directly represents the practical/operational application of performance measurement, especially in the context of pediatric emergency care.

In summary, this targeted analysis confirms that, beyond the organization of the network into four distinct thematic clusters, the four central terms function as interconnected nodes-bridges, which ensure the coherence of the field: quality of care (“quality of health care”) constitutes the conceptual core, “benchmarking” the methodological bridge to clinical outcomes, “organization and management” the bridge to the administrative/institutional framework, and “quality improvement” the bridge to operational/emergency implementation.

### 3.6. Bibliographic Coupling Analysis

**Figure 8:**
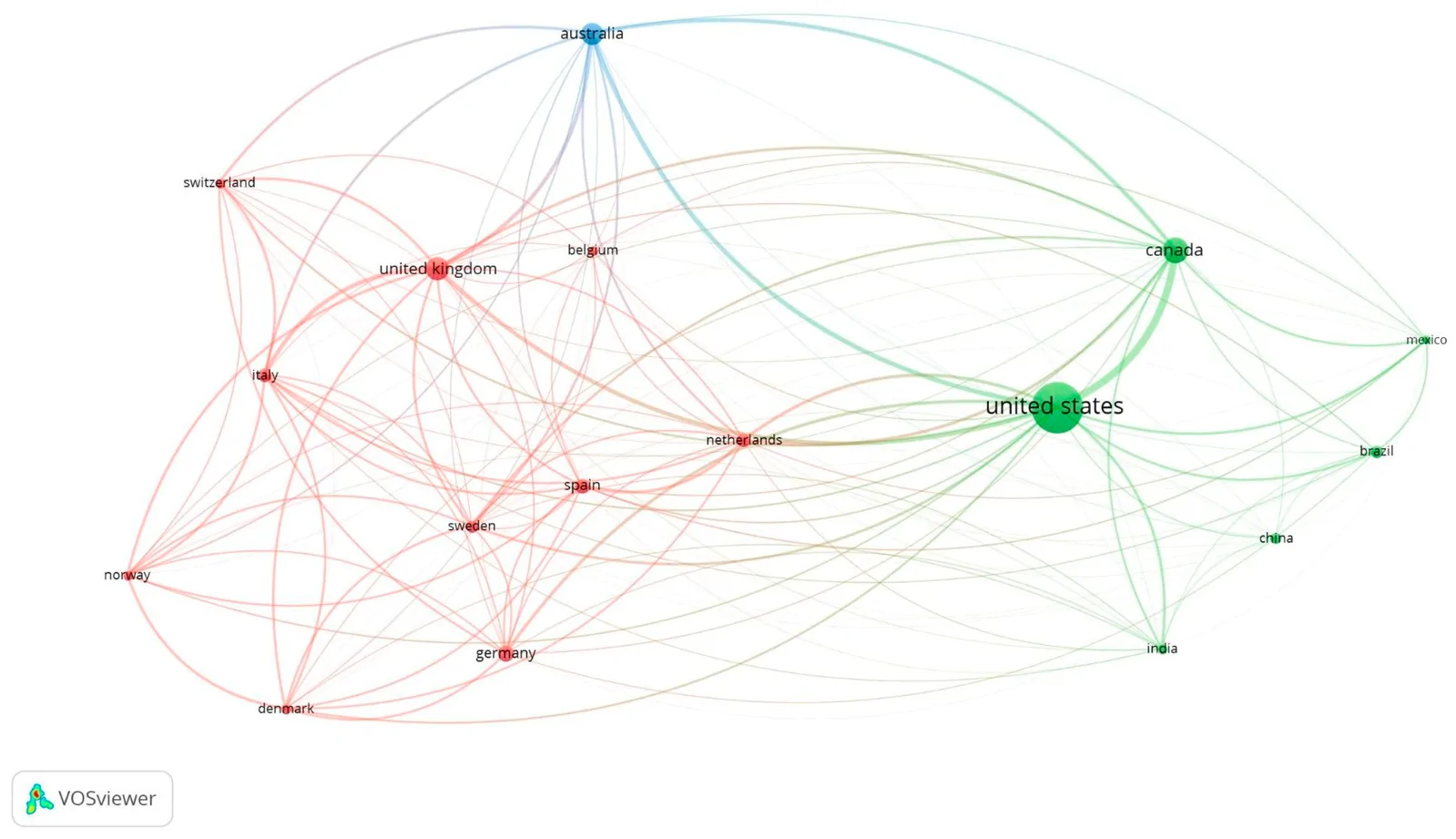
Network Visualization Analysis (Bibliographic Coupling Analysis).

To examine similarities in the reference patterns of the retrieved literature across countries, a bibliographic coupling analysis was performed at the country level using the full counting method. Bibliographic coupling reflects similarity between countries based on the references cited by their publications and should not be interpreted as a direct measure of research collaboration or co-authorship. A minimum threshold of ten (10) documents per country was set, a criterion that was met by 17 out of a total of 126 countries in the dataset. All 17 countries that met the criterion were included in the analysis, without additional filtering based on the number of citations. The analysis returned a network of three distinct country clusters (Figure 8). The document counts reported in this bibliographic coupling analysis correspond to the values generated by VOSviewer and may differ slightly from the descriptive country/territory publication counts reported directly by Scopus in Section 3.1 because the two analyses rely on different counting procedures.

Cluster 1 (red, 10 countries)—European cluster: Belgium, Denmark, Germany, Italy, Netherlands, Norway, Spain, Sweden, Switzerland and the United Kingdom. The United Kingdom emerges as the dominant node of the cluster (62 documents, 1565 citations, total link strength 3055), followed by Italy (23 documents, TLS 1939) and the Netherlands (22 documents, TLS 1778). This cluster represents a coherent European bibliographic linkage network, indicating that European countries share a high degree of common bibliographic references with each other.

Cluster 2 (green, 6 countries)—Transcontinental/English-speaking-emerging cluster: Brazil, Canada, China, India, Mexico and the United States. The United States is the absolute dominant node not only of the cluster but of the entire network (290 papers, 7128 citations, total link strength 4984), followed by Canada (71 papers, TLS 3556). The strong US-Canada connection reflects a shared bibliographic base of references, while the inclusion of countries such as Brazil, China and India in the same cluster indicates an increasing international coupling of emerging research with the dominant American research stream.

Cluster 3 (blue, 1 country)—Australia forms a separate cluster (56 papers, 827 citations, total link strength 2715). In the visualization, Australia is positioned between the European and transcontinental clusters and displays bibliographic coupling links with countries in both groups. This position indicates similarities in cited-reference patterns with both clusters; however, visual placement alone does not establish a formal bridging or mediating role. Overall, the bibliographic coupling analysis identifies distinct geographical patterns in the similarity of cited-reference profiles, with a predominantly European cluster and a transcontinental cluster dominated by the United States and Canada, while Australia forms a separate cluster. These patterns should be interpreted as similarities in the bibliographic foundations of the countries’ publications rather than as evidence of direct international research collaboration.

## 4. Discussion

A particularly interesting finding of the present study concerns the distribution of publications by subject area. Although performance measurement and management are concepts that traditionally originate from the field of administrative science and organizational management, the retrieved literature is strongly concentrated in medical and clinical subject areas. This finding suggests that performance management in child and adolescent health services has developed mainly as a field of clinical evaluation, quality improvement and measurement of care outcomes, rather than as an independent subject of administrative science. The extremely limited presence of publications in the Business, Management and Accounting category may indicate a comparatively limited representation of explicitly managerial perspectives within the retrieved literature. However, this pattern should be interpreted cautiously. Scopus subject-area classifications provide information on the disciplinary classification of the retrieved publications but are not equivalent to a direct content-based classification of individual studies and may partly reflect journal-level indexing. In addition, the composition of the retrieved literature may have been influenced by the health-focused terminology incorporated into the search strategy, including several quality-oriented terms. Therefore, in the absence of direct content coding of the retrieved publications, these findings should be regarded as indicating a possible research gap in explicitly administrative and managerial performance perspectives rather than confirming their absence from the broader field of child and adolescent healthcare.

Nevertheless, relevant pediatric and adolescent healthcare studies demonstrate that performance-related research also extends to organizational, operational, and resource-related dimensions. A qualitative study by Goff et al. [18] examined organizational characteristics associated with high-performing pediatric primary care practices, including teamwork, leadership, practice structures, and quality-improvement tools. A service evaluation by Fuggle et al. [30] examined efficiency in Child and Adolescent Mental Health Services (CAMHS), including waiting times and patient flow. More recently, Ibrahim et al. [31] examined health-facility capacity and technical efficiency in adolescent healthcare services, including operational, managerial, adaptive, and leadership capacity. In parallel, Muzigaba et al. [32] developed a core set of pediatric quality-of-care indicators intended to support monitoring, accountability, learning, and improvement across health-system levels. Taken together, these studies illustrate the broader range of organizational, operational, efficiency, and measurement dimensions that may warrant further integration and investigation within pediatric and adolescent healthcare performance-management research.

It is worth noting that the exact term “performance measurement”, although central to the title and research question of the present study, is not included in the final network of 111 keywords, as its literal frequency of occurrence (6 occurrences) falls short of the inclusion threshold (15). This absence from the thresholded network should not be interpreted as evidence that the concept itself is absent from the literature or that it is necessarily represented through other terminology. Rather, it reflects the methodological effect of the selected occurrence threshold on the terms included in the network. Although related terms such as “quality indicator”, “benchmarking”, “total quality management” and “quality improvement” were more prominent in the co-occurrence analysis, their presence alone does not establish that they function as substitutes for the concept of performance measurement across the retrieved literature. Any such conceptual relationship should therefore be interpreted cautiously and in conjunction with the broader literature rather than inferred solely from keyword frequency. At this point, it is worth clarifying the conceptual relationship between the terms that dominated the keyword network (quality indicator, benchmarking, quality improvement, quality of health care) and the broader field of performance measurement and management, which is the subject of this study. This distinction is not arbitrary: as Persaud and Nestman [10] point out, simple performance measurement differs substantially from performance management, as the latter presupposes the existence of clear mechanisms for converting feedback from indicators into specific action strategies; the same authors emphasize that quality improvement is precisely the element that “fulfills the administrative dimension of performance measurement”, transforming it into an integral part of a performance management framework. In this light, quality indicators function as the measurement layer of performance management, benchmarking as the mechanism for interpretation and comparison, and quality improvement as the management action that results from them—three stages of a single cycle “measurement → comparison → action”, which constitutes the core of any performance management framework. The operation of this cycle is also empirically documented by the pilot implementation of the World Health Organization’s PATH tool (Performance Assessment Tool for quality improvement in Hospitals) in 37 hospitals in six countries, where successful implementation was based precisely on the integration of performance indicators, international benchmarking, and self-improvement mechanisms within a single performance management framework (Groene et al. [33]). Based on the above, the prominence of the terms “quality indicator”, “benchmarking” and “quality improvement” indicates that these concepts occupy an important position within the retrieved literature and are conceptually related to broader processes of performance measurement and management, as discussed above. However, the present keyword analysis alone does not establish that these terms constitute empirical substitutes for “performance measurement”, nor does their prominence by itself confirm the absence of broader strategic management frameworks in this research field.

The keyword co-occurrence analysis revealed four distinct thematic clusters, which correspond to four relatively autonomous but interrelated research axes: quality of care and its methodological assessment tools, clinical outcomes and hospital experience, administrative/institutional framework, and emergency care operational procedures. The consistent presence of the term “quality of health care” as the most interconnected node in all stages of the analysis (co-occurrence, density, targeted analysis) confirms the role of quality of care as a unifying concept that bridges the clinical, administrative and operational dimensions of performance in health services—a finding consistent with the experience of implementing performance measurement systems such as the Performance Measurement System (PMS) of Tuscany, where quality indicators based on the patient experience were systematically combined with administrative data to define strategic objectives, incentives and management tools at the health system level (Nuti et al. [34]). This case demonstrates in practice how quality of care can function as a common language between clinicians, administrators and policymakers.

Regarding the temporal stratification of the field, the overlay analysis revealed that the administrative/institutional cluster consistently displays an older average publication time than the clinical and operational clusters. A possible explanation for the more recent average publication years observed for clinical and operational terms may be related to broader developments in the digitalization and data-driven organization of healthcare. The widespread adoption of electronic health records and interoperable health information exchange has substantially increased the availability and exchange of electronic clinical data (Barker et al. [35]). In parallel, learning health system approaches use routinely collected data to continuously monitor and improve healthcare outcomes, supported by clinical analytics and digital infrastructures (Lim et al. [36]; Somerville et al. [37]). These developments may have contributed to a research environment in which clinical outcomes, measurement, and operational improvement receive increasing attention. However, this represents a plausible interpretation of the observed bibliometric pattern rather than a causal relationship demonstrated by the present analysis.

The country-level bibliographic coupling analysis identified distinct geographical patterns in the similarity of cited-reference profiles, including a predominantly European cluster and a transcontinental cluster dominated by the United States and Canada, while Australia formed a separate cluster. These groupings reflect similarities in the references cited by publications from the included countries rather than direct research collaboration. Accordingly, the observed structure should be interpreted as a descriptive bibliographic pattern and not as evidence of geographical research “bipolarity” or of a formal bridging role for Australia. A separate country-level co-authorship analysis would be required to investigate international research collaboration directly. Finally, the apparent underrepresentation of explicitly administrative and managerial performance frameworks specifically focused on pediatric/adolescent care within the retrieved literature is reinforced by specific examples in the international literature. The “Tiers of Service” framework developed in British Columbia, Canada (Waibel et al. [38]) is an indication that, when frameworks for organizing pediatric services are systematically developed, they focus mainly on the operational planning of service provision (levels of care, division of responsibilities) and not on performance management in the sense of strategic indicators, costs or organizational capacity. A similar picture emerges from a recent rapid review of care standards for pediatric services (Barber et al. [39]), which confirms that existing “levels of service” frameworks focus on roles and responsibilities, while the review itself explicitly calls for further research on how to implement, adapt and evaluate these frameworks in practice. In a similar vein, an empirical study of primary health care systems in Malawi [40] showed that, even when formal performance management policies (Performance Measurement and Management systems) exist, their implementation is hampered by weak interconnection between levels of management, incomplete dissemination of priorities and limited utilization of performance information by decision-makers—a finding that, although it concerns primary care systems in general and not exclusively pediatric care, reveals the practical difficulties of implementing performance management frameworks in health systems, especially in developing contexts.

Taken together, the findings indicate that the literature retrieved through the present search is predominantly clinically oriented, whereas explicitly administrative and managerial performance perspectives are less prominent. This pattern may point to a potential research gap; however, it should not be interpreted as definitive evidence that such perspectives are absent from the broader literature. The observed thematic distribution may partly reflect the construction of the search strategy itself, which included quality-oriented terms such as “quality indicator” and “benchmarking”, while other potentially relevant managerial concepts were not explicitly incorporated. Accordingly, the predominance of clinical quality terminology identified in the present analysis should be interpreted as a characteristic of the retrieved dataset rather than as conclusive evidence of the conceptual structure of the entire field.

## 5. Study Limitations and Further Research

The present study presents certain limitations that should be considered when interpreting its findings.

First, the data were exclusively extracted from the Scopus database. Although Scopus has particularly extensive coverage of the international scientific literature in the field of health sciences, publications listed exclusively in other databases, such as Web of Science, PubMed or Dimensions, were not included in the analysis. The use of a single database carries the risk of partial coverage of the relevant literature, especially publications from regional or non-English-speaking sources, which may not be fully covered by individual international databases. The absence of a comparative analysis with Web of Science or PubMed may therefore introduce database-related publication and coverage bias, potentially affecting the observed distribution of publications, keywords, thematic patterns, and geographical representation. Consequently, the thematic, temporal, and geographical patterns identified in the present analysis should be interpreted as reflecting the literature indexed in Scopus rather than as an exhaustive representation of all publications in the field. Future studies could examine the use of multiple databases in comparison or combination, in order to ensure broader and more representative coverage of the field.

Second, the results of any bibliometric study are inevitably influenced by the search strategy adopted, as well as by the terminology used by the authors themselves. Despite the use of a proximity operator to improve the accuracy of the search and limit irrelevant results, it cannot be ruled out that some relevant publications were not retrieved, due to variations in the terminology used or in the formulation of concepts. In addition, no formal title-and-abstract eligibility screening was applied to the 643 retrieved records, as the study was designed as a bibliometric mapping of the literature retrieved by the predefined Scopus query rather than as a systematic review of individually screened studies. Consequently, despite the use of the W/10 proximity operator to improve search specificity, some retrieved records may use terms such as “performance”, “evaluation”, or “assessment” in clinical, diagnostic, technical, or treatment-related contexts rather than in the organizational performance-management sense. This possibility should be considered when interpreting the observed predominance of clinical and quality-related terminology.

More specifically, the search strategy included quality-oriented terms such as “quality indicator” and “benchmarking”, whereas other potentially relevant concepts within the broader domain of performance management, such as management control, healthcare governance, accountability, efficiency, resource allocation, productivity, value-based care, and hospital performance, were not explicitly included as search terms. Consequently, the composition of the retrieved dataset may have favored literature using clinical quality terminology. This potential search-strategy bias should be considered when interpreting the observed predominance of clinical and quality-related concepts in the bibliometric network. The present findings therefore characterize the literature retrieved through the predefined search strategy and should not be interpreted as demonstrating that the broader field of pediatric and adolescent healthcare performance management is inherently or exclusively clinically oriented.

Third, the keyword co-occurrence analysis method, like any keyword-based bibliometric mapping technique, has inherent limitations in terms of fully capturing the conceptual content of a research field. The keywords assigned by authors or databases reflect the linguistic and terminological choice of a subset of stakeholders (authors, database editors), which is not necessarily uniform nor fully representative of the overall conceptual content of each publication. Furthermore, the application of a minimum threshold of occurrences (in the present study, ≥15) de facto excludes lower frequency terms, which may carry significant conceptual weight but have not yet gained widespread diffusion in the literature—a typical example is the phrase “performance measurement” itself, which, as highlighted in the Discussion, did not meet this threshold despite its centrality to the subject of the study. The co-occurrence analysis, therefore, captures a partial, terminologically “popular” image of the field, and should not be interpreted as a complete mapping of its conceptual content.

In addition, the bibliometric mapping was conducted exclusively with VOSviewer, and no validation using an alternative bibliometric technique or software was performed. The sensitivity analysis using alternative keyword occurrence thresholds (≥10 and ≥20) provided an assessment of the extent to which the network structure depended on the selected inclusion threshold; however, this analysis should be regarded as a robustness check rather than as an independent validation of the clustering solution. Future studies could complement VOSviewer-based mapping with alternative bibliometric techniques or software to examine the reproducibility and stability of the identified thematic structure.

Fourth, the interpretation of some of the findings of the Discussion—particularly regarding the temporal differences between administrative and clinical research terminology and the geographical patterns observed in the country-level bibliographic coupling analysis—remains exploratory, as no study was found in the international literature that explicitly examines these patterns in this specific field. These interpretations should therefore be treated as indicative directions for further investigation rather than as documented conclusions.

In terms of further research, the findings of this study highlight several directions that are worth exploring in future studies. First, there is a need to develop and empirically evaluate performance management frameworks specifically tailored to the specificities of pediatric/adolescent care, which would incorporate dimensions of strategy, cost, organizational capacity, and human resources, in addition to purely clinical quality assessment. Existing pediatric service organization frameworks, such as the “Tiers of Service” [38], provide a useful basis for service delivery planning, but the question remains how such frameworks could be extended to incorporate systematic indicators of administrative performance. At the same time, as highlighted by a recent review of care standards [39], there is a need for research on how to implement, localize, and systematically evaluate existing “service level” frameworks in practice. Finally, empirical studies in different health systems—particularly in low- and middle-income settings, where, as the study by Makwero et al. [40] in Malawi showed, the implementation of performance management systems encounters significant barriers in practice—would contribute to understanding the conditions under which performance management frameworks can be effectively implemented in child and adolescent health services, in both developed and developing health systems.

## 6. Conclusions

This study provides a systematic bibliometric mapping of 643 Scopus-indexed publications on performance measurement and management in child and adolescent healthcare services from 1990 to 2026. Research production increased markedly after 2010, while keyword co-occurrence analysis identified four interconnected thematic areas: quality of care and methodological assessment tools, clinical outcomes and hospital experience, administrative/institutional frameworks, and emergency care operational procedures. “Quality of health care” emerged as the central connecting concept across these areas. The overlay analysis also showed that administrative and institutional terms were associated with relatively older publications, whereas clinical and operational terms were associated with more recent publications. Geographically, research output was concentrated particularly in the United States, Canada, the United Kingdom, and Australia.

Overall, the findings indicate a potential research gap in the administrative and managerial dimensions of performance measurement in pediatric and adolescent healthcare. The literature retrieved through the present search is strongly concentrated in clinical and health-related areas, while explicitly management-focused perspectives appear comparatively limited within the retrieved dataset. The findings therefore suggest a potential direction for future research involving the development and empirical evaluation of comprehensive performance-management approaches that integrate clinical quality with managerial and strategic dimensions and are specifically adapted to the characteristics of pediatric and adolescent healthcare services. This proposed direction represents an interpretation of the bibliometric findings rather than a management framework directly derived from or empirically evaluated by the present analysis. Accordingly, the suitability, feasibility, and effectiveness of any specific performance-management framework in pediatric and adolescent healthcare settings require direct empirical evaluation in future research.

## Figures and Tables

**Table 1:** Thematic clusters of keywords (keyword co-occurrence analysis, VOSviewer).

Cluster	No Terms	Dominant Terms (Occurrences)	Thematic Orientation
1 (red)	33	quality of health care (299), quality indicator (188), total quality management (97), practice guideline (64)	Quality of care, standards, methodological assessment tools (Delphi)
2 (green)	32	benchmarking (124), outcome assessment (92), pediatric hospital (78), risk factor (46)	Clinical outcomes, risk factors, hospital care
3 (blue)	29	quality control (81), organization and management (60), health care delivery (57), health care policy (39)	Administration, health policy, organizational/institutional framework
4 (yellow)	17	procedures (86), quality improvement (85), emergency department (49), emergency health service (37)	Emergency care, operational procedures, quality improvement

**Table 2:** Temporal distribution of indicative terms (Overlay Analysis).

Term	Cluster	Average Year of Publication	Occurrences
Standard	3	2006.6	43
Medicaid	3	2008.6	19
Organization and management	3	2011.8	60
Quality control	3	2012.3	81
Delphi study	1	2019.4	39
Registries	2	2020.1	21
Surgery	2	2020.5	20
Therapy	4	2021.2	17
Hospital emergency service	4	2021.5	21
Clinical outcome	2	2023.3	17

## Data Availability

No new data were created or analyzed in this study. Data sharing is not applicable to this article.

## Supplementary Materials

The following supporting information can be downloaded at: https://www.mdpi.com/article/10.3390/healthcare14162598/s1, File S1: VOSviewer thesaurus file used for keyword harmonization, including term merging and exclusion of thematically uninformative terms; File S2: Original VOSviewer network export containing the final keyword set included in the primary co-occurrence analysis (minimum occurrence threshold ≥ 15) and the associated network and bibliometric attributes. File S3: Original VOSviewer country-level bibliographic coupling network export containing the country-level network attributes and bibliographic coupling links used in the analysis.
